# Layering of a health, nutrition and sanitation programme onto microfinance-oriented self-help groups in rural India: results from a process evaluation

**DOI:** 10.1186/s12889-021-12049-0

**Published:** 2021-11-20

**Authors:** Laili Irani, Janine Schooley, Supriya, Indrajit Chaudhuri

**Affiliations:** 1 Population Council, Zone 5A, Ground Floor, India Habitat Centre, Lodi Road, New Delhi, 110003 India; 2 Project Concern International, 5151 Murphy Canyon Rd, Suite 320, San Diego, CA 92123 USA; 3 Project Concern International, 38, Okhla Phase 3 Rd, Okhla Phase III, Okhla Industrial Area, New Delhi, 110020 India

**Keywords:** Self-help groups, Health, nutrition and sanitation programming, Maternal, neonatal and child health, Rural India

## Abstract

**Background:**

The state of Bihar has been lagging behind Indian national averages on indicators related to maternal and child health, primarily due to lack of knowledge among mothers of young children on lifesaving practices and on where to seek services when healthcare is needed. Hence, the JEEViKA Technical Support Programme was established in 101 blocks to support the state rural livelihood entity, JEEViKA, in order to increase demand for and link rural families to existing health, nutrition and sanitation services. Programme activities were geared to those engaged in JEEViKA’s microfinance-oriented self-help groups. These groups were facilitated by a village-based community mobilizer who was trained on health, nutrition and sanitation-related topics which she later shared in self-help group meetings monthly and during ad hoc home visits. Further, a block-level health, nutrition and sanitation integrator was introduced within JEEViKA to support community mobilizers. Also, indicators were added into the existing monitoring system to routinely capture the layering of health, nutrition and sanitation activities.

**Methods:**

A process evaluation was conducted from August–November 2017 which comprised of conducting 594 quantitative surveys with community mobilizers, from program and non-programme intervention blocks. Linear and logistic regressions were done to capture the association of at least one training that the community mobilizers received on knowledge of the topics learned and related activities they carried out.

**Results:**

Community mobilizers who had received at least one training were more likely to have higher levels of knowledge on the topics they learned and were also more likely to carry out related activities, such as interacting with block-level integrators for guidance and support, routinely collect data on health, nutrition and sanitation indicators and spend time weekly on related activities.

**Conclusions:**

Successful integration of health, nutrition and sanitation programming within a non-health programme such as JEEViKA is possible through trainings provided to dedicated staff in decentralized positions, such as community mobilizers. The findings of this evaluation hold great promise for engaging existing non-health, nutrition and sanitation systems that are serving vulnerable communities to become partners in working towards ensuring stronger health, nutrition and sanitation outcomes for all.

## Background

**Fig. 1:**
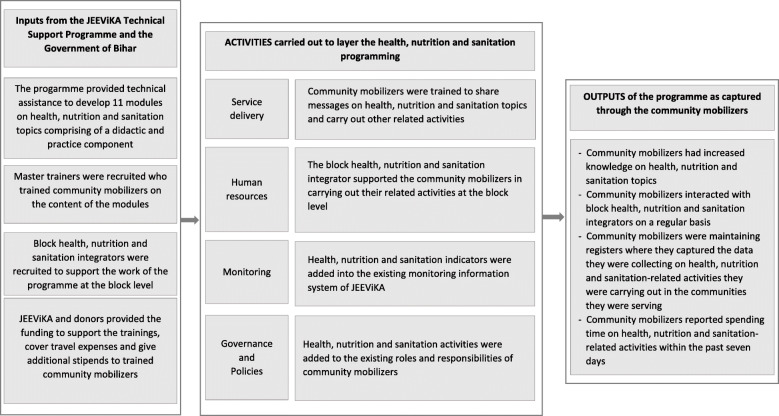
The process evaluation framework for the assessment of the training programme on intended outcomes among the community mobilizers

India has made great strides in reducing maternal and infant mortality rates [1–3]. However, disparities exist across states with northern populous states, such as Bihar, having the highest burdens of maternal and child mortality (274 maternal deaths per 100,000 live births as compared to the national average of 197; 48 infant deaths per 1000 live births as compared to the national average of 44; and, 70 under-five child deaths per 1000 live births as compared to the national average of 55) [4]. The primary causes of these mortalities are from preventable factors such as a lack of knowledge on best maternal and child health and nutrition practices and absence of information on where to seek appropriate healthcare services [5, 6]. In light of this evidence, the government of Bihar from 2010 onwards began engaging with various development partners to co-develop and implement a package of essential and locally relevant health and nutrition-related interventions that would address the delivery of appropriate healthcare services as well as create a demand for such services in communities. On this account, the government tested a pilot intervention to layering a health, nutrition and sanitation (HNS) programme onto newly formed microfinance-oriented self-help groups^1^ in 64 blocks across eight districts^2^ [8]. Led by Project Concern International, a global development organization, one of the approaches of the pilot project entailed sharing of information on maternal, neonatal and child HNS practices during weekly-held self-help group meetings using a participatory learning approach; these discussions were led by a trained health volunteer. An outcome evaluation showed an improvement in key maternal, neonatal and child health outcomes, such as institutional delivery, practice of skin-to-skin care and exclusive breastfeeding, as a result of the intervention [8, 9]. Enthused by these results and other similar trials in India and Nepal that had shown strong evidence of the positive association of women’s groups on maternal, neonatal and child health indicators, the state-led entity responsible for managing and supporting government-led microfinance-oriented self-help groups, JEEViKA, scaled up the intervention to 101 blocks across 8 districts with support from the Bill and Melinda Gates Foundation, World Bank and Project Concern International [7, 10–12]. Hence, the JEEViKA Technical Support Programme was created in 2015. One of the key initial components of the programme was to train the grassroots functionaries of JEEViKA, called community mobilizers,^3^ to learn about healthy maternal, neonatal and child HNS practices so that they in turn could share these practices with other women (who had young children) in the community using a participatory learning approach [13–15]. A process evaluation, commissioned by JEEViKA and the Bill and Melinda Gates Foundation, was led by independent evaluators and carried out in the second year of the programme’s implementation in order to capture some learnings on the association of the trainings on the work the community mobilizers were carrying out. This information would further be used to refine the programme and guide future scale-up within the state and beyond. Hence, the objective of this paper is to share the results of a process evaluation that captured the association of HNS trainings on both the knowledge and the related activities the community mobilizers carried out to assist mothers with young children in the communities they were serving (Fig. 1).

The hypothesis of this evaluation was that those community mobilizers who were trained on HNS topics were more likely to have increased knowledge of correct practices related to HNS and were also more likely to carry out activities that were linked to spreading this information with their group members, than those who did not receive any trainings. The theory of change that this study was built around was that if community mobilizers were trained on HNS topics, then not only will their knowledge on correct HNS-related practices increase, but they would be able to effectively share this information with women in self-help groups. This would in turn result in these women practising healthy HNS behaviours and seeking the appropriate preventive and curative services, when needed. This intervention was uniquely placed to have a significant impact on maternal, neonatal and child outcomes as it was being implemented through self-help groups where the women from historically marginalized communities were coming together to empower themselves economically and socially in order to become productive members of their families and society.

There are few documented examples that describe the impact of such training programs established within government departments in order to build institutional capacity. One such example is presented by Sgaier et al. (2014) who describe the association of a technical support unit that was established in the Indian state of Karnataka to support the National AIDS (acquired immunodeficiency syndrome) Control Programme [16]. This unit enhanced the managerial and technical capacities of the programme to support its intended beneficiaries. Other investments and efforts that have been evaluated have looked at the implementation of development programs across related sectors, such as nutrition and early childhood development [17, 18]. These articles describe the challenges of integrating multisectoral programs and highlight the need for high-level political buy-in coupled with the need for inherent capacity within the institution to take up new tasks coupled with adequate funding. Our paper adds to this existing literature by further documenting the association of layering an HNS-focused intervention onto a non-health state-led entity. This research notes the system-level changes that occurred at the grassroots in order to successfully layer this new programming onto an existing well-established microfinance-oriented programme.

### Intervention

Inputs to the programme were developed with the intention to build JEEViKA’s capacity to layer HNS interventions onto the existing microfinance-related activities that were already ongoing (Fig. 1). One of the inputs included developing and introducing modules on key HNS practices that would increase the knowledge of women belonging to self-help groups on the practice of healthy maternal, neonatal and child HNS behaviours. These modules were developed using a human-centred design approach and the quality of the content and teaching was ensured through handholding support, mentoring, supervision and review by the staff of Project Concern International. The modules included an orientation module highlighting the importance of HNS and covered other topics such as early initiation of breastfeeding, complications during pregnancy, antenatal care, birth preparedness, basic new-born care, complications that could arise among mothers and new-borns, family planning, elements of good maternal and child nutrition, need for proper sanitation, among others.

Master trainers, taught these modules to the community mobilizers; these trainers were recruited by Project Concern International and reported to JEEViKA staff at block level. The only cadre of JEEViKA staff recruited to specifically support this programme were the block health, nutrition and sanitation integrator, henceforth referred to as the block integrator, who were hired and trained by Project Concern International. JEEViKA bore the financial costs for: the HNS trainings, printing modules and other materials, travel and daily allowances for the block integrators when visiting the field, and monthly stipends (300 Indian rupees/4.5 United States dollars) to the community mobilizers who had received the training and as a result would be expected to carry out additional tasks. In addition, the Bill and Melinda Gates Foundation funded Project Concern International to support the JEEViKA Technical Support Program.

A primary activity that was carried out to layer the programme within JEEViKA was training community mobilizers on key maternal, neonatal and child HNS messages. During these trainings, they were also informed of the additional tasks they would undertake to layer HNS programming into their daily activities related to self-help groups. Classroom instructions were enhanced through sharing of real-life examples, such as case studies and supplementary materials. The community mobilizers were supported by the block integrator whose primary role included organizing trainings on HNS modules across the block, providing post-training support to the community mobilizers, monitoring the implementation of the modules and coordinating with various JEEViKA departments across the 101 blocks where the programme was being implemented. HNS indicators were added into existing monitoring systems within JEEViKA to ensure that HNS-related data would be collected, analysed and used routinely across the organization. The job descriptions of community mobilizers were revised to include additional responsibilities for them to carry out HNS-related activities.

JEEViKA Technical Support Program’s efforts were geared towards increasing the capacity of community mobilizers to layer HNS-related activities onto existing and evolving self-help groups and related community initiatives. Hence, the outputs of the intervention capture the change in knowledge of community mobilizers on HNS topics they learned during the trainings. The other outputs measured included other related activities community mobilizers were expected to carry out following their first training, namely regularly interacting with block integrators, routinely collecting data on indicators that were capturing the implementation of HNS-related activities within groups and communities, and spending time on any HNS-related activity within the past week.

## Methods

### Research design

The process evaluation was carried out from August–November 2017. Using a cross-sectional study design, ten blocks were sampled, each from a different district of Bihar; seven came from blocks where the JEEViKA Technical Support Programme was being implemented and the remaining three from non-intervention blocks. Ethical clearance was sought from the independent evaluators’ institutional review board, the Population Council Institutional Review Board.

In consultation with Project Concern International, the management and organizational sustainability tool was modified and used to design a quantitative survey tool that was administered to the community mobilizers [19, 20]. The questionnaire was designed specifically for this study to capture the strategic, structural and system-level changes the community mobilizers reported that they had adopted in order to implement the additional set of activities they were expected to carry out following their training (Additional file 1).

### Study sample and data collection

For this evaluation, 594 community mobilizers were interviewed across the 10 blocks (418 from programme blocks and 176 from non-programme blocks). Any community mobilizer who was currently employed by JEEViKA was eligible to be part of the survey. Villages were randomly selected from each of the blocks and only one community mobilizer per village was invited to participate in the survey. If they were not available, another community mobilizer in the same village was approached.

The interviews were conducted in Hindi using structured tools, carried out by trained investigators using a mobile-based data entry application, CSPro 6.3 [21]. A group of quality assurance monitors were attached to each data collection team. About one-third of the surveys were spot checked to ensure quality control. The data were then analysed using Stata 13 [22].

There were four key dependent variables of interest that were captured during the surveys. The first indicator captured the level of knowledge of the community mobilizers on HNS topics. In order to measure this indicator, community mobilizers were asked 34 questions related to knowledge and awareness of correct practices on HNS. For questions with binary responses, those who responded to the questions correctly received a single score. For questions with multiple responses, each respondent received a fractional score depending on the number of correct responses they gave. For example, if a question had four potential correct responses, each correct response elicited 0.25 score, up to a maximum score of one for that question. Further, all the 34 knowledge-related questions were summed up to form a continuous variable. In order to capture the second dependent variable, community mobilizers were asked if they had interacted with the block integrator in the past year, captured as a dichotomous variable. To measure the third dependent variable, community mobilizers were asked if they had reported on HNS indicators in their routine monitoring registers over the past month; these indicators were numbers of: self-help group meetings where HNS messages were shared, village health, nutrition and sanitation days—held monthly— in which self-help group members participated, and households visited by community mobilizers to check on a pregnant woman/child under the age of 2. Community mobilizers compiled these data in HNS-designated forms and registers; the block integrators in turn prepared monthly reports to share with other JEEViKA staff. In order to capture the fourth dependent variable, community mobilizers listed all the self-help group-related activities they had carried out in the last 7 days. These included conducting a self-help group meeting where HNS messages were shared or visiting the home of a self-help group member to share HNS-related messages, filling in the health and nutrition register, among others.

**Fig. 2:**
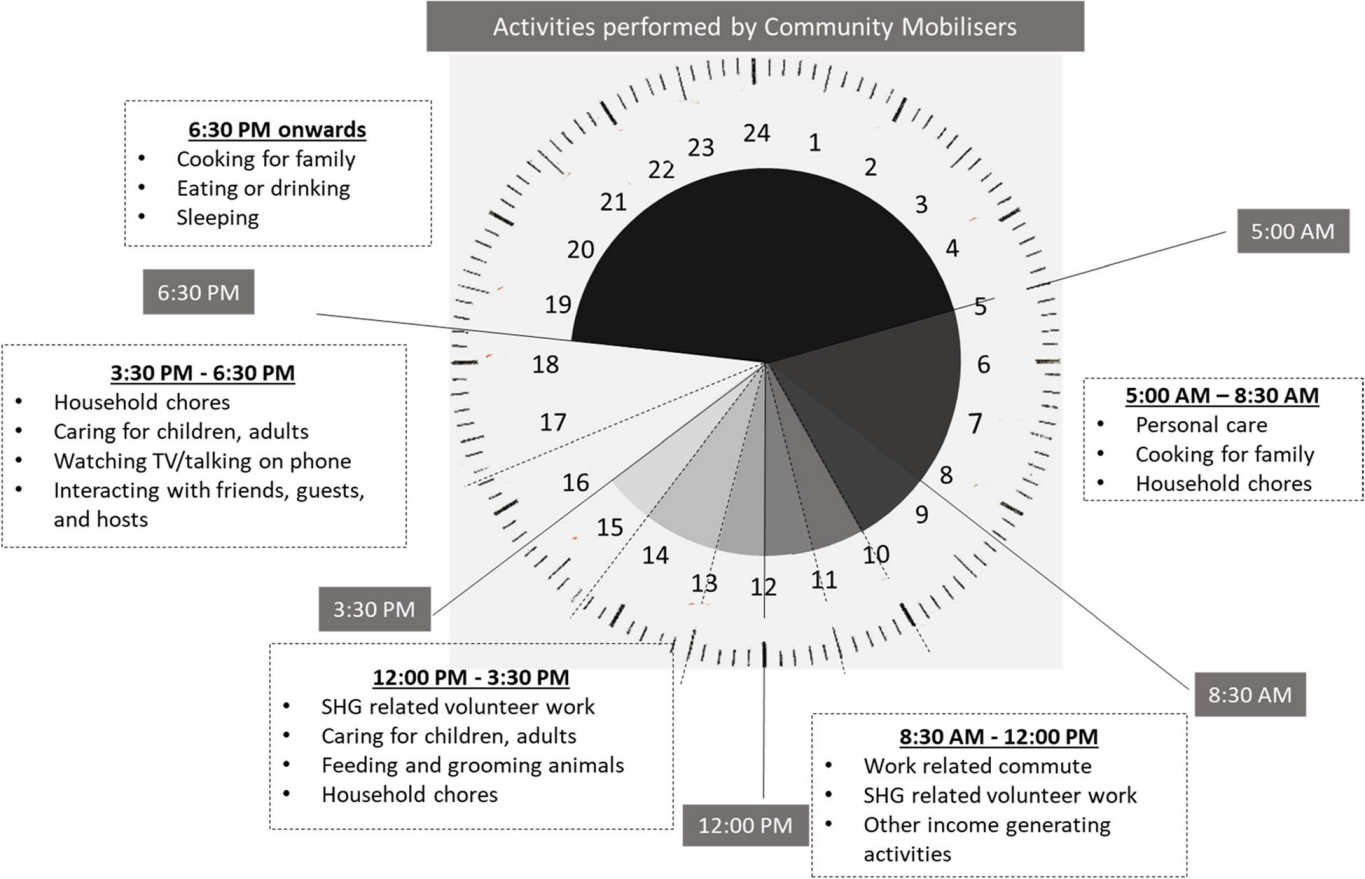
Average time spent by community mobilizers (CMs) on various activities over the past 24 h

The time spent by the community mobilizers on key tasks and activities over the course of the week was also captured, comparing the results with those who received HNS training and those who did not. In addition, the interviewers took a time log of the activities the community mobilizers conducted in the 24 h preceding the interview in order to get a sense of how community mobilizers carried out their self-help group functions and other activities over the course of their day (Fig. 2).

The key independent variable of interest was whether the community mobilizers reported receiving a training on an HNS topic in the past year or not. All community mobilizers from blocks where the programme was being implemented and who had received at least one training were considered as having ‘received training on HNS topics.’

Other covariates were also captured and assessed in determining the association of training on the implementation of HNS activities by the community mobilizers. These indicators included: the age of the respondent (in years, categorized into three groups (18–24, 25–34, 35/older)); gender (male, female); religion (Hindu, others); caste (scheduled castes/scheduled tribes, other backward castes, others); education (0–8 years of schooling, 9–10 years (high school), 11–12 years (higher secondary school), 13/more); marital status (currently married, other statuses); mobile phone ownership (personal, shared with the family); type of mobile phone (brick, feature, smartphone); average duration of association with JEEViKA in months; and, average number of hours the community mobilizer worked in a day. Basic sociodemographic indicators were controlled for in the multivariate analyses as indicators such as education and age have been known to be associated with levels of knowledge and performance related to capacity building efforts [23, 24]. Furthermore, length of association with an organization and the number of hours worked were also known to be associated with performance [25–27].

### Analysis plan

A distribution of the respondents by the key background characteristics is presented in Table 1. Next, chi-squared tests were conducted to capture the distribution of background characteristics among those who had received training versus those who had not (Table 1). Further, the knowledge scores, for each individual knowledge-related question, of those respondents who had received training were compared with the scores of those respondents who had not received any training; t-tests were run for questions with multiple correct answers and two proportion z-tests were run for questions with binary responses (Table 2).

**Table 1:** Distribution of background characteristics of community mobilizers, by receipt of training on HNS topics

Background Characteristics		Percentage (%)		
		Total (***N*** = 594)^**a**^	Did not receive HNS training^**b**^	Received HNS training^**b**^
**Study Arm**	Programme blocks	70.4	11.0	89.0^d^
	Non-programme blocks	29.6	100.0	0.0
**Age**	18–24 years	33.3	50.0	50.0
	25–34 years	39.9	33.3	66.7
	35 years and above	26.8	27.7	72.3^d^
	Mean (Standard Deviation)	29.2 (7.9)	27.1 (7.1)	30.4 (8.1)
**Gender**	Male	0.8	80.0	20.0^c^
	Female	99.2	37.1	62.9
**Religion**	Hindu	95.8	32.0	68.0
	Others	4.2	37.6	62.4
**Caste**	Scheduled castes/ Scheduled tribes	20.2	33.3	66.7
	Other backward castes	68.9	37.4	62.6
	Others	10.9	44.6	55.4
**Education**	0–8 years	11.1	22.7	77.3
	9–10 years (high school)	33.7	38.5	61.5
	11–12 years (higher secondary school)	32.2	38.2	61.8
	13+ years	23.1	41.6	58.4
**Marital status**	Other marital status	19.0	39.8	60.2
	Currently married	81.0	36.8	63.2
**Own a mobile phone**	Personal	89.9	56.7	43.3^d^
	Shared	10.1	35.2	64.8
**Type of mobile phone**	Basic brick phone	75.8	38.2	61.8
	Featured phone	16.2	33.3	66.7
	Smart phone	6.4	26.3	73.7^c^
**Duration of association**	Duration of association with JEEViKA in months (mean, SD)	31 (21)	22 (16.2)	37^c^ (21.8)
**Work duration**	Weekly working hours (mean, SD)	18.4 (13.8)	17.2(13.3)	19.2 (14.1)

^a^column distribution by characteristics

^b^chi-squared tests were done for each characteristic comparing row percentages

^c^denotes chi square test at 5% level of significance

^d^denotes chi square test at 1% level of significance

**Table 2:** Distribution of knowledge scores of community mobilizers, by receipt of training/not

**A. Knowledge Indicators** (Average scores)A.	**Average knowledge score of CMs** (For each indicator, knowledge score ranges from 0 to 1)		
	**Total** **(*N*** **= 594)**	**Did not receive HNS training** **(*N*** **= 222)**	**Received HNS training** **(*N*** **= 372)**
1. Importance of registering a pregnancy	0.5	0.3	0.6^b^
2. Tests to be conducted during antenatal care check-ups	0.5	0.4	0.7^b^
3. Steps that can be taken to prepare delivery for birth (home/hospital)	0.6	0.3	0.7^b^
4. Benefits of institutional delivery	0.4	0.3	0.5^b^
5. Nutritional requirements of a pregnant woman/ lactating mother in comparison to a non-pregnant woman	0.4	0.2	0.5^b^
6. Elements of proper nutrition during pregnancy	0.5	0.4	0.6^b^
6. Steps to be carried out immediately following the birth of a new-born	0.2	0.1	0.3^b^
8. Elements to be included in post-natal check-ups of the new-born	0.3	0.2	0.4^b^
9. Elements comprising skin to skin care	0.7	0.5	0.8^b^
10. Steps taken to ensure clean umbilical cord care	0.3	0.1	0.4^b^
11. Potential health complications to look out for during the first month of birth	0.2	0.1	0.2^b^
12. Steps that can be taken to make a child’s food nutritious and energy dense	0.3	0.2	0.4^b^
13. Services provided at the Anganwadi Centre	0.3	0.2	0.4^b^
14. Services provided during the monthly-held village health, nutrition and sanitation day	0.3	0.1	0.4^b^
**B. Knowledge Indicators** (Proportions)	**Proportion of CMs who answered correctly to binary knowledge indicators**		
	**Total** **(*N*** **= 594)**	**Did not receive HNS training** **(*N*** **= 222)**	**Received HNS training** **(*N*** **= 372)**
1. Pregnancy should be registered during the first trimester	80.6	68.5	87.9^b^
2. At least three antenatal check-ups should be conducted during a pregnancy	28.6	9.5	40.1^b^
3. A pregnant woman should receive two or more anti-tetanus injections during pregnancy	84.0	72.5	90.9^b^
4. A pregnant woman should take 100 or more iron-folic acid tablets during pregnancy	72.1	46.9	87.1^b^
5. The health institution is the preferred location for a delivery	90.4	85.1	93.6^b^
6. A pregnant woman should be given information on who to call for assistance during an emergency	93.4	86.0	97.9^b^
7. Respondent knows the emergency number for the ambulance service	76.4	50.9	91.7^b^
8. Respondent is aware of the availability of 24-h ambulance service	70.5	59.5	77.2^b^
9. Knowledge that a new-born baby should be examined by a trained healthcare professional within the first hour of birth	94.6	92.4	96.0
10. Breastfeeding of the new-born should be initiated in the first hour of birth	95.1	90.5	97.9^b^
11. A baby should not be given a bath for the first 72 h of life	73.9	55.9	84.7^b^
12. Pregnant women should consume tricolored food items daily	76.3	46.9	93.8^b^
13. The minimum normal weight of a new-born is 2500 g	68.7	65.8	70.4
14. Infants under six months of age should not be given water	82.5	68.5	90.9^b^
15. Semi-solid foods should be initiated at six months of age	93.9	91.9	95.2
16. Foods that can be initiated after six months of age include water, animal/formula milk, semi-solid or solid food like khichdi			
17. Children between the ages of 6–24 months should only receive semi-solid foods	74.8	77.0	73.4^a^
18. ORS treatment should be given during diarrhoea	66.2	52.7	74.2^b^
19. The government provides the new mother an incentive of INR 1400 under Janani Evam Bal Suraksha Yojana (JBSY) on delivering at a government health facility	97.8	96.4	98.7
20. Annaprashan Divas is held on 19th of every month	78.1	62.2	87.6^b^

^a^denotes t-test/two proportion z-test at 5% level of significance

^b^denotes t-test/two proportion z-test at 1% level of significance

t-tests were run for indicators that were captured through average scores due to multiple correct responses (part A) and two proportion z-tests were run for questions having only dichotomous responses (part B)

Further analysis was conducted into determining what self-help group-related tasks and activities community mobilizers spent their time on. The proportion of community mobilizers who reported spending any time carrying out key tasks and activities was noted. Further, t-tests were conducted to compare the average time spent (in hours) by the community mobilizers on these various tasks and activities over the past 7 days, by those who had received training versus those who had not (Table 3).

**Table 3:** Distribution of key tasks and activities performed by community mobilizers in past 7 days and average time spent on these activities

Key tasks and activities	Percentage of community mobilizers who report carrying out the specific activity in past 7 days (***N*** = 594)	Average time spent on these tasks and activities in past seven days (in hours)		
		Total (***N*** = 594)	Did not receive HNS training (***N*** = 222)	Received HNS training (***N*** = 372)
Conducting meetings where health, nutrition and sanitation modules are rolled out	6.1	1.3	0.9	1.4^b^
Maintaining health and nutrition register	23.7	1.5	1.4	1.5^b^
Conducting a meeting on non-health topics	40.1	11.9	13.5	11.1
Maintaining books of record	43.3	2.1	2.6	1.9
Training SHG members on non-health topics	23.7	2.3	2.0	2.3^b^
Preparing micro-plan for self-help groups	19.9	2.1	2.0	2.1^a^
Facilitating bank linkages of self-help groups	12.8	0.9	1.2	0.8
Forming new self-help groups	12.0	3.0	4.6	2.1
Facilitating opening new bank accounts for members	11.6	0.5	0.6	0.4
Attending village organization, cluster and related administrative meetings	6.7	0.5	0.8	0.5^a^
Resolving conflicts and disagreements among self-help group members	2.9	0.3	0.3	0.3

^a^denotes t-test at 5% level of significance

^b^denotes t-test at 1% level of significance

Bivariate distribution of each of the independent variables were compared by each of the four dependent variables of interest individually and tested for significance using chi-squared tests (Table 4). Next, multivariate regression models were run to capture the association of training on each of the four dependent variables separately, in order to identify key factors associated with the uptake of HNS knowledge and behaviours (Table 5). The independent variables were included in each of the analyses as control variables. A linear regression was run to measure the association of training on average knowledge scores and logistic regressions were run to capture the association of training on the other three dependent variables of interest, namely interacting with a block integrator, maintaining HNS registers and having spent any time on HNS activities in the past week. Two multivariate models were run. In model I, only the key independent variable of interest, i.e., whether received training or not, was included against each of the dependent variables. In model 2, all the other sociodemographic characteristics were added to each of the analyses. The robustness of each of the multivariate models was tested by adding each of the covariates individually to each model one at a time, by eliminating outliers and rerunning the analyses, as well as bootstrapping the analyses up to 500 repetitions [28]; all tests determined that the linear and logistic regression models were robust for the analyses carried out.

**Table 4:** Bivariate analysis showing association of outcome indicators by background characteristics

Background Characteristics		Knowledge and Awareness score	Interacting with block integrator	Maintain HNS registers	Spent time on HNS activity in past week
		Average score	Yes	Yes	Yes
		21.0 (*N* = 594)	54.4	59.9	26.9
**Received training on HNS**	Yes	23.7	82.0^b^	88.4^b^	36.6^b^
	No	16.5	8.1	12.2	10.8
**Study arm**	Community mobilizers in programme blocks	22.9	76.8^b^	82.8^b^	34.2^b^
	Community mobilizers in non-programme blocks	16.5	1.1	5.7	9.7
**Age**	18–24 years	19.2	41.9^b^	50.5^b^	23.7
	25–34 years	21.6	59.9	62.8	29.5
	35+ years	22.4	61.6	67.3	27.0
	Mean (SD)	–	30.3 (7.99)	30.1 (8.04)	29.4 (7.31)
**Gender**	Male	18.5	20.0	20.0	0.0
	Female	21.0	54.7	60.3	27.2
**Caste**	Scheduled castes/Scheduled tribes	21.6	55.0	61.7	27.5
	Other backward castes	21.1	56.2	59.9	26.4
	General	19.6	41.5	56.9	29.2
**Education**	0–8 years of schooling	22.3	60.6	69.7	33.3
	Completed 9th/10th grade (high school)	20.9	55.5	54.0	25.5
	Completed 11th/12th grade (higher secondary school)	20.8	53.4	61.8	25.7
	Completed 13+ years of schooling	20.7	51.1	61.3	27.7
**Marital status**	Currently married	19.3	51.3	59.3	26.6
	Currently not married	21.4	55.1	60.1	27.0
**Own a mobile phone**	Personal	18.7	56.5^a^	48.3	20.0
	Shared	21.3	35.0	61.2	27.7
**Type of mobile phone**	Basic brick phone	20.8	54.9^b^	57.8^b^	27.3
	Featured phone	21.2	54.2	68.8	26.0
	Smart phone	23.0	60.5	73.7	31.6
**Length of association**	Average months of association with JEEViKA	–	4.4 (1.39)	4.4 (1.38)	4.4 (1.31)
**Work duration**	< 18 h	20.7	52.2	53.5^b^	19.9^b^
	≥18 h	21.3	56.6	66.3	34.0

^a^denotes chi square test at 5% level of significance

^b^denotes chi square test at 1% level of significance

**Table 5:** Multivariate regression showing effect of HNS training, and other characteristics on HNS knowledge and activities (bootstrap, 500 replications)

		Model A: Knowledge Scores	Model B: Interaction with BHNSI	Model C: Maintain HNS register	Model D: Spent time on HNS activity in past week
		Beta coef. (SE)	OR (95% CI)	OR (95% CI)	OR (95% CI)
**Model I**					
**R-square**		0.374	0.242	0.330	0.036
**Received training on HNS** (Ref: No)		1.05^b^ (0.05)	1.70^d^ (1.55–1.87)	2.08^d^ (1.85–2.36)	1.15^d^ (1.09–1.22)
**Model II**					
**R-square**		0.428	0.304	0.372	0.064
**Received training on HNS** (Ref: No)		0.96^b^ (0.06)	1.61^d^ (1.41–1.83)	2.03^d^ (1.63–2.54)	1.15^d^ (1.07–1.23)
**Age** (Ref: 18–24 years)	25–34 years	1.13^a^ (0.46)	1.77^c^ (1.01–3.12)	1.20 (0.66–2.18)	1.24 (0.70–2.19)
	35+ years	1.28^a^ (0.56)	1.11 (0.59–2.09)	1.13 (0.53–2.42)	0.99 (0.52–1.88)
**Caste** (Ref: Scheduled castes/ Scheduled tribes)	Other backward castes	−0.33 (0.45)	1.22 (0.73–2.05)	0.95 (0.52–1.74)	0.98 (0.58–1.65)
	General	−0.79 (0.63)	0.90 (0.36–2.26)	1.75 (0.72–4.23)	1.43 (0.70–2.94)
**Education** (Ref: 0–8 years of schooling)	Completed 9th/10th grade (high school)	−1.05 (0.59)	0.86 (0.44–1.71)	0.48 (0.22–1.03)	0.62 (0.33–1.19)
	Completed 11th/12th grade (higher secondary school)	−0.24 (0.60)	0.97 (0.47–1.98)	0.98 (0.43–2.21)	0.62 (0.33–1.18)
	Completed 13+ years of schooling	−0.25 (0.63)	0.86 (0.38–1.94)	0.99 (0.42–2.34)	0.67 (0.34–1.34)
**Marital status** (Ref: Single)	Currently married	1.17^a^ (0.55)	0.72 (0.39–1.32)	1.00 (0.49–2.03)	0.90 (0.50–1.63)
**Own a mobile** (Ref: Shared mobile)	Personal mobile	0.73 (0.56)	2.06^c^ (1.03–4.12)	1.09 (0.58–2.03)	1.19 (0.54–2.60)
**Duration of association:** Average months of association with JEEViKA		0.03^b^ (0.01)	1.04^d^ (1.02–1.05)	1.02^d^ (1.01–1.04)	1.00 (0.99–1.01)
**Weekly work duration (Ref: < 18 h)**	18 h or more	0.12 (0.35)	0.90 (0.60–1.37)	1.66^c^ (1.07–2.55)	2.10^d^ (1.40–3.14)

^a^denotes beta coefficient at 5% level of significance

^b^denotes beta coefficient at 1% level of significance

^c^denotes odds ratio at 5% level of significance

^d^denotes odds ratio at 1% level of significance

## Results

Of the 418 community mobilizers interviewed from programme blocks, 89% (372) had received at least one HNS training till the time of data collection. Table 1 presents the distribution of respondents who received trainings versus those who did not, by key background characteristics of the community mobilizers. Majority of the community mobilizers were 25–34 years old (39.9%), women (99.2%), belonged to the Hindu religion (95.8%) and were from other backward castes (68.9%). Furthermore, majority had completed nine or more years of schooling (89.0%), were married (81%) and owned a mobile phone (89.9%) which was primarily a basic brick phone (75.8%). On average, community mobilizers had been associated with JEEViKA for around 2.5 years and worked 18 h a week on self-help group-related activities, amounting to an average of 3.6 hours/day for 5 days a week. Results from chi-squared tests comparing the characteristics of those who had received at least one training versus those who had not suggested that community mobilizers who were older women, owned a smart phone and had been associated with JEEViKA for more than 3 years were more likely to have received a training on HNS topics than their counterparts.

Community mobilizers who had received at least one training had more knowledge of healthy practices related to HNS behaviours across most indicators than those who did not receive any training (Table 2). Of those trained, almost two-thirds (61%) had received training on four/more topics. The most common topics the community mobilizers recalled being trained on were the introductory module on the importance of HNS (70.3%), antenatal care practices (54%), basic new-born care (53.2%), appropriate immunization (25%), adequate child nutrition (21.8%) and correct sanitation practices (15.9%).

The key tasks and activities that the community mobilizers reported spending time on over the course of a week included: conducting self-help group meetings, sharing HNS messages when possible, maintaining books of records documenting savings and other indicators, preparing micro-plans for the groups, and facilitating bank linkages for the members (Table 3). Community mobilizers who had received an HNS training spent more time carrying out HNS-related activities, such as capturing HNS indicators, and sharing HNS messages in self-help group meetings and through home visits, than those who had not been trained. A 24-h time use analysis of the previous day showed that community mobilizers generally carried out their self-help group-related activities in the morning and early afternoon, i.e., between 8:30 AM and 3:30 PM (Fig. 2). This pattern did not significantly differ for those who were trained on HNS topics versus those who were not and there were no other substantial differences across geographies or sociodemographic characteristics of the community mobilizers. Time use analysis shows that for the rest of the day, the community mobilizers were pre-occupied with household responsibilities and a small proportion of them were also engaged in other income generating activities, such as non-farm economic activities, raising animals, etc.

Among the key outcomes of interest, community mobilizers had an average knowledge score of 21.0 (out of 34) (Table 4). The average score for knowledge was higher among those who had received training (23.7) versus those who had not (16.5). Just over half the community mobilizers (54.4%) had interacted with the block integrator over the past year, three-fifths (60%) had recorded HNS indicators over the past month, and around a quarter (26.9%) had carried out HNS-related activities during the past week. Community mobilizers that interacted with the block integrators and maintained HNS registers were more likely to be under the age of 25 and own their own personal brick/feature phone as compared to older community mobilizers who did not own their own phone.

Multivariate analyses suggest that community mobilizers who received HNS trainings had greater knowledge on HNS topics than those who had not received any training (β = 0.96; SE = 0.06; *p* < 0.001) (Table 5). Further, community mobilizers who had participated in at least one HNS training had significantly higher odds of interacting with a block integrator (OR = 1.61; CI = 1.41–1.83; *p* < 0.001), maintaining registers where HNS indicators were captured (OR = 2.03; CI = 1.63–2.54; *p <* 0.001) and carrying out HNS-related activities in the past week (OR = 1.15; CI = 1.07–1.23; *p* < 0.001). Also, knowledge around HNS topics increased with age and duration of association with JEEViKA. Community mobilizers who were 25–34 years and owned their own mobile phones had greater odds of interacting with a block integrator than younger community mobilizers and those who did not own their own phone. For every 1 month increase in association with JEEViKA, the odds of interacting with a block integrator (OR = 1.04; CI = 1.02–1.05; *p* < 0.001) and capturing HNS indicators (OR = 1.02; CI = 1.01–1.04; *p* < 0.001) increased. Similarly, those who worked more than the average 18 h a week had higher odds of maintaining HNS registers (OR = 1.66; CI = 1.07–2.55; *p* < 0.005) and spending time on HNS activities over the past week (OR = 2.10; CI = 1.40–3.14; *p <* 0.001) than those working fewer hours. The higher r-squares in Model II for each of the multivariate analyses as compared to Model I confirms the robustness of the multivariate models.

## Discussion

Community mobilizers were the driving force for layering of HNS-related activities within self-help groups and across JEEViKA, in general. They were trained on HNS topics which resulted in a significant increase in the community mobilizers’ knowledge on healthy behaviours and practices related to maternal, neonatal and child HNS. The approach employed by JEEViKA is corroborated by existing evidence suggesting that regular members from a community with a non-health background can be trained to share basic health and nutrition preventive messages [29–31]. However, our study went further to show the association of layering an HNS-focused intervention within a non-HNS state-led entity, JEEViKA and identified the system-level changes that were carried out in order to implement this additional program. It addressed the shortcomings that previous literature has noted of a limited high-level management support coupled with the need for adequate capacity building and funding [17, 18]. This programme overcame those barriers as the senior management team at JEEViKA was highly supportive of this intervention in building capacity at the grassroots and was assisted by other partners and donors. As a result, it was able to overcome the challenges often seen when integrating multisectoral programs, in this case being the integration of a health program onto a non-health public entity. Going forward, JEEViKA would benefit from engaging and coordinating with HNS-related community institutions, existing government schemes and related departments (such as the health department and integrated child development services).

The knowledge that community mobilizers gained on HNS topics was translated into action by them sharing the new information they learned in at least one self-help group meeting per group, every month, and in visits they made to homes of self-help group members where there was a pregnant woman or mother of a young child residing. Other activities they carried out included connecting with the block integrator to regularly discuss updates on their activities. Community mobilizers also periodically collected and reported on HNS indicators which were collated and reviewed at regular intervals across the organization signalling a united interest and commitment to HNS layering. Potential key motivators for JEEViKA staff to pick up new additional tasks and perform them with zeal and enthusiasm could be strengthened coordination and regular use of data as noted in previous literature [32–36]. Further, longer association with JEEViKA was associated with greater odds of carrying out HNS layered activities, such as interacting with block-level staff and maintaining the HNS register. These findings are consistent with previous studies showing that increased association within an existing programme led to increased self-conscientiousness to carry out activities related to one’s job and to stay motivated [25, 26].

An analysis of how community mobilizers spent their time over the course of a single day suggested that community mobilizers appeared to have flexible working hours and managed to carry out their work-related duties in addition to their household tasks and economic activities they were pursuing. These findings are consistent with other studies that have shown that flexible working hours for women assists them to stay committed and satisfied with their job [37, 38]. A comparison of time spent on self-help group-related activities over the course of a week showed that when community mobilizers layered HNS-related activities onto their existing responsibilities, they replaced the time they were spending on non-HNS activities by HNS activities thus contributing the same time to their overall responsibilities towards their groups. This suggests that the overall time burden of community mobilizers is not increasing due to additional HNS-related tasks and responsibilities. As the total time available to a community mobilizer is constant, it appears that if more tasks were to be given to community mobilizers, they would need to prioritize their tasks. Hence, in order to ensure that the new HNS component of the programme stays integrated within JEEViKA and the existing mandate of the microfinance efforts that the public entity is engaged in are also carried out, it may need to consider appointing a dedicated cadre of staff at a more decentralized level, beyond the block integrator to support the work of the community mobilizers.

Finally, layering of interventions that share HNS messages within community platforms such as the self-help groups shows great promise. As JEEViKA establishes new groups across the state, introducing trainings on HNS topics to newly inducted community mobilizers, building on lessons learned to date, will assist in integrating such programming further. Lastly, programs looking to integrate a new technical component into an existing government led programme or platform originally designed for another purpose can explore utilizing a similar approach.

### Future studies

More research is needed to study the impact of layering such programming onto self-help groups at scale with improving behavioural outcomes in women, their children and families. Such studies could further capture the effect of each of the components of the intervention on behavioural outcomes while measuring the cost effectiveness of such an approach as compared to other interventions attempting to change behaviours around HNS. Future impact studies could also help us understand how the economic and social empowerment of women, nurtured within self-help groups, and coupled with such programming on HNS really catalysed long term change at the individual, family and community level. Further, as the JEEViKA Technical Support Programme evolved to implement additional interventions, such as organizing community events where HNS messages were shared with the larger rural audience, and community mobilizers and villages who had enthusiastically carried out the programme were felicitated publicly, the impact evaluation would hope to measure the association of these additional components of the intervention on the program’s intended outcomes.

### Limitations

A limitation of the study was that data were collected at one time point with no prior information on the knowledge, attitudes and competencies of the community mobilizers. This limitation was addressed by interviewing community mobilizers from districts and blocks where the programme was not ongoing. We did not collect data from another state as sociodemographic differences across states are stark coupled with different approaches to running state-led self-help group programs. Another limitation of this study was that data were self-reported by community mobilizers who had received training and who were implementing HNS activities. This was countered through deliberate questioning and probing conducted by senior research staff who asked objective quantifiable questions related to knowledge on HNS topics and other related activities that the community mobilizers were implementing on the ground.

## Conclusion

In summary, the training of grassroots functionaries, the community mobilizers, led to their increased knowledge on HNS topics as well as greater implementation of related activities. Layering HNS activities into an existing non-health programme requires the key protagonists, in this case community mobilizers, to gain adequate knowledge of related topics and be supported through continued accompaniment coupled with significant time and effort put in by all. With receptivity among the community mobilizers to be trained, and acceptability among self-help group women to receive health-related information from community mobilizers, integrating HNS messaging within self-help groups poses a great opportunity for filling the void that exists at the grassroots in terms of limited knowledge on healthy maternal, neonatal and child HNS practices. Layering such a programme within self-help groups, which are inherently empowering women economically and socially, may thus hold the key to better health outcomes for women and their children.

## Data Availability

The datasets generated and/or analyzed during the current study are available in the Harvard Dataverse repository, 10.7910/DVN/PKETRB.

## Abbreviations

AIDS: Acquired immunodeficiency syndrome
HNS: Health, nutrition and sanitation

## Footnotes

1 A self-help group is comprised of 8–12 women from marginalized communities and living close by [7]. They meet weekly to save money and borrow funds from each other and banks at low interest rates.

2 Bihar state is administratively divided into 38 districts which are further subdivided into 534 blocks.

3 A community mobilizer is the grassroots functionary of JEEViKA and is responsible for managing 10–12 self-help groups in a village. She convenes weekly held self-help group meetings, manages the financial books of each group, links the groups to local banks and is the liaison of the groups with JEEViKA.
